# Comparative evaluation of two dose-volume histogram prediction tools for treatment planning: Treatment planning quality and dose verification accuracy

**DOI:** 10.1016/j.tipsro.2024.100297

**Published:** 2024-12-13

**Authors:** Shoma Nakano, Motoharu Sasaki, Yuji Nakaguchi, Takeshi Kamomae, Kanako Sakuragawa, Yuto Yamaji, Hitoshi Ikushima

**Affiliations:** a School of Health Sciences, Tokushima University, Tokushima, Tokushima 770-8503, Japan; b Graduate School of Biomedical Sciences, Tokushima University, Tokushima, Tokushima 770-8503, Japan; c Toyo Medic Co., Ltd., Chiyoda-ku, Tokyo 162-0813, Japan; d Radioisotope Research Center, Nagoya University, Nagoya, Aichi 464-8602, Japan; e Department of Radiology, Nagoya University Graduate School of Medicine, Nagoya, Aichi 466-8550, Japan; f Department of Radiological Technology, Tokushima University Hospital, Tokushima, Tokushima 770-8503, Japan

**Keywords:** Verification, RapidPlan, PlanIQ, Volumetric-modulated arc therapy

## Abstract

**Purpose:**

This study aims to compare treatment plans created using RapidPlan and PlanIQ for twelve patients with prostate cancer, focusing on dose uniformity, dose reduction to organs at risk (OARs), plan complexity, and dose verification accuracy. The goal is to identify the tool that demonstrates superior performance in achieving uniform target dose distribution and reducing OAR dose, while ensuring accurate dose verification.

**Methods:**

Dose uniformity in the planning target volume, excluding the rectum, and dose reduction in the OARs (the rectum and bladder) were assessed. The validation included point-dose measurements with an ionization chamber dosimeter and gamma analysis of dose distributions. Monitor units were calculated to evaluate plan complexity.

**Results:**

PlanIQ provided superior dose uniformity, with improvements in the dose homogeneity index compared with RapidPlan. RapidPlan was more effective in reducing OAR doses, particularly in the rectum, with significant reductions at various dose levels. Dose verification showed no significant differences between the two tools. However, PlanIQ showed a smaller mean difference between the calculated and measured doses and a slightly better dose distribution match with less variability than RapidPlan.

**Conclusions:**

RapidPlan was more effective at reducing OAR doses, whereas PlanIQ achieved better dose uniformity and lower plan complexity. Both tools performed similarly in terms of dose verification accuracy, with PlanIQ showing a slight advantage in dose-distribution matching. The choice of planning tool depends on the primary treatment goal, whether it is to reduce the OAR doses or improve the target dose uniformity.

## Highlights

• PlanIQ provided superior dose uniformity compared with RapidPlan.
• RapidPlan was more effective in reducing OAR doses at various dose levels.
• Dose verification showed no significant differences between the two tools.
• PlanIQ showed a smaller mean difference between the calculated and measured doses.
• Both tools performed similarly in terms of dose verification accuracy.

## Introduction

Volumetric-modulated arc therapy (VMAT), a leading technique in high-precision radiotherapy, has recently been shown to improve treatment outcomes because of its superior dose distribution compared with three-dimensional conformal radiation therapy [1]. The radiation intensity in VMAT within the treatment field is modulated using a multileaf collimator (MLC) while the gantry rotates. This allows for a high dose distribution that is both uniform and conforms to the target, while simultaneously minimizing exposure to surrounding organs at risk (OARs) [2], [3], [4].

However, a significant challenge with VMAT-based treatment planning is that the quality of plans can vary depending on the facility and experience of the planner [5]. This issue has been addressed by the use of tools like RapidPlan (Varian Medical Systems, Palo Alto, CA, USA) [6], a knowledge-based planning (KBP) system, and the treatment planning quality assurance (QA) software PlanIQ (Sun Nuclear, Melbourne, FL, USA) [7].

RapidPlan uses historical patient treatment data to build models that predict achievable dose-volume histograms (DVHs) for new patient targets and OARs [8]. Several studies have demonstrated that RapidPlan can improve the quality of treatment planning by reducing variability between planners and centers [9], [10], [11]. In contrast, PlanIQ employs Feasibility DVH (FDVH) to predict the potential for dose reduction to each OAR in advance based on computed tomography (CT) and contour data [8]. Studies report that PlanIQ enhances the treatment planning quality [7], [12]; however, no report has confirmed whether treatment plans developed using either tool meet the QA standards required for patient irradiation with linear accelerators.

Consequently, we aimed to evaluate the differences in dose uniformity to the target and dose reduction to the OARs between RapidPlan and PlanIQ to compare the quality of the treatment plans. Subsequently, we aimed to perform a pretreatment validation of patient plans created using both tools, to assess the accuracy of the dose calculations in the treatment plans.

## Materials and Methods

### Patient Enrollment and treatment planning Overview

This retrospective study included 12 patients with prostate cancer who underwent VMAT at our institution. These patients were randomly selected from a group of 111 patients treated between January 2015 and December 2020 using the random number generation feature in Microsoft Excel 2016 (Microsoft Corporation, Redmond, WA, USA). Detailed patient information is provided in Table 1. The study was conducted in accordance with the ethical principles outlined in the Declaration of Helsinki and in accordance with the ‘Recommendations for the Conduct, Reporting, Editing and Publication of Academic Research in Medical Journals’. All procedures involving human subjects were carried out in compliance with relevant legal and institutional guidelines and were approved by the University of Tokushima Hospital Ethics Committee (approval number 3434). Informed consent was obtained from all participants prior to their inclusion in the study, and their privacy rights were strictly observed.

**Table 1:** Patient characteristics.

	Structure		
	PTV-R [cc]	Rectum [cc]	Bladder [cc]
Patient 1	111.79	50.40	392.45
Patient 2	92.63	25.75	91.75
Patient 3	103.48	28.39	78.95
Patient 4	85.82	33.86	114.53
Patient 5	144.70	37.93	155.06
Patient 6	95.58	38.91	288.09
Patient 7	84.09	23.79	203.17
Patient 8	144.43	26.54	95.01
Patient 9	189.57	35.45	169.65
Patient 10	155.33	41.35	104.85
Patient 11	101.95	33.33	173.65
Patient 12	95.91	41.16	129.05

All treatments were performed using a TrueBeam linear accelerator (Varian Medical Systems, Palo Alto, CA, USA) operated at an X-ray energy of 10 MV. VMAT was selected as the treatment modality and involved two complete arcs per session. The prescribed radiation dose was 2 Gy per fraction for 39 sessions, resulting in a total radiation dose of 78 Gy. Treatment planning was performed using the Eclipse treatment planning system (TPS) (Varian Medical Systems, Palo Alto, CA, USA) version 16.1.0, with dose calculations based on the analytic anisotropic algorithm (AAA). The collimator angles were set to 30° and 330°, and a dose calculation grid size of 2.5 mm × 2.5 mm × 2.5 mm was employed.

The contours included the planning target volume excluding the rectum (PTV-R), clinical target volume (CTV), rectum, and bladder. The contouring methodology documented previously was used [2]. The dose constraints associated with these contours are provided in Table 2.

**Table 2:** Dose constraints for treatment plans.

Structure	Constraint
CTV	D100 % > 99.5 %
PTV-R	D95 % = 78 Gy
Rectum	V40 Gy ≤ 50 %
	V60 Gy ≤ 25 %
	V70 Gy ≤ 15 %
	V75 Gy ≤ 5 %
Bladder	V40 Gy ≤ 50 %
	V60 Gy ≤ 25 %

### Development of the RapidPlan model using PlanIQ

The RapidPlan model used in this study was constructed using the FDVH tool available in PlanIQ. The FDVH is a tool that categorizes a DVH into four regions: red (impossible), orange (difficult), yellow (challenging), and green (likely achievable). The classification relies on the F-values defined by Ahmed et al. for each region: F-value = 0 for impossible regions, 0 < F-value ≤ 0.1 for difficult regions, and 0.1 < F-value ≤ 0.5 for challenging regions [13]. The model used here is based on the validated work by Masumoto et al. and is currently suitable for clinical implementation [8].

### Treatment planning with RapidPlan

DVH predictions based on patient anatomy were generated in the treatment-planning phase using RapidPlan. These predictions were then used to automatically suggest optimal dose distribution parameters, forming the basis for creating a planning template. The optimization parameters used in this study are listed in Table 3.

**Table 3:** Optimization parameters used in treatment planning.

Structure	Vol[%]	Dose[Gy]	Priority
CTV			
Upper	0.0	82.0	Generated
Lower	100.0	80.0	Generated
PTV-R			
Upper	1.0	81.8	Generated
Upper	0.0	82.0	Generated
Lower	97.0	80.0	Generated
Lower	100.0	78.0	Generated
Bladder			
Upper	30.0	40.0	Generated
Lower	10.0	65.0	Generated
Line（preferring OAR）	Generated	Generated	Generated
Rectum			
Upper	10.0	58.0	Generated
Upper	20.0	38.0	Generated
Upper	60.0	18.0	Generated
Line（preferring OAR）	Generated	Generated	Generated

### Treatment planning with PlanIQ

CT images and contour data were first transferred from the Eclipse TPS system to PlanIQ for treatment planning. The dose calculation grid size and energy selection used for FDVH calculations were set to 2.5 mm × 2.5 mm × 2.5 mm and 10 MV-X, respectively, with the assumption that the final treatment plan would be created in Eclipse with reference to the FDVH. In our previous study, treatment planning in Eclipse, guided by FDVH referencing, was successfully improved by targeting an achievable region with an F value of ≤ 0.1 [8]. Accordingly, a similar approach was adopted for the treatment planning in this study.

### Assessment of treatment plans created by RapidPlan and PlanIQ

The treatment plans were evaluated by directly extracting dose metrics from the DVH for each endpoint. The evaluated parameters included doses to the CTV and PTV-R at various percentiles (D2%, D50%, and D98%), as well as dose-volume data for the rectum and bladder. Additionally, the homogeneity index (HI), which indicates dose uniformity, was calculated for PTV-R using the following equation:

$$\mathrm{HI}=\frac{D_{2\%}-D_{98\%}}{D_{50\%}}$$

Furthermore, the calculated monitor units (MUs) were examined as indicators of treatment plan complexity. Although other metrics are available for evaluating treatment plan complexity, this study focused on MU because of their simplicity and ease of calculation.

### Verification Methods

Point-dose and dose distribution verifications were performed to verify the treatment plan. Point-dose verification was performed using an ionization chamber dosimeter, TN31014 (PTW, Freiburg, Germany), and an electrometer (Ramtec SMART; Toyo Medic Co., Ltd., Tokyo, Japan). Given that the treatment plan involved fractionated irradiation for prostate cancer VMAT, a high-dose area, and a low-dose gradient were observed within approximately 1 cm of the isocenter. Therefore, dosimetry was conducted only at the isocenter and not at multiple points. The ionization chamber dosimeter used for point dose verification has a short diameter of 2.0 mm, a long diameter of 5.0 mm, and a volume of 0.015 cc; the grid size for dose calculation in the TPS is 2.5 mm × 2.5 mm × 2.5 mm, and the ionization chamber dosimeter occupies two voxels; therefore, no dose difference occurs. Readings of the calculated values were performed using point doses. Point-dose verification was assessed based on the difference between the dose calculated by the TPS and the actual measured dose. The dose calculated using the TPS was considered the reference value. An RT-2300 Cylinder phantom (R-TECH Co., Ltd., Tokyo, Japan) was used for point-dose verification.

A Delta4 PT array detector (ScandiDos AB, Uppsala, Sweden) was used to verify the dose distribution. γ-analysis was employed for dose distribution verification using the Delta4 PT. The doses were evaluated in three categories: 1 mm/1%, 2 mm/2%, and 2 mm/3% of the absolute dose. Furthermore, the dose threshold for all the assessment criteria was set to 10 %.

### Assessment based on research guidelines for treatment planning

Hansen et al [14] have developed guidelines for treatment planning studies. These guidelines, known as the Guidelines for Treatment Planning Research, aim to enhance the quality of research in this field through a structured maturity assessment framework that includes numerical scoring. The scoring system covers a wide range of criteria, based on the typical structure of a scientific article. It begins with an outline of the study’s design and development, followed by a critical discussion of the results and a conclusion. There are a total of 76 scoring items. A score of 189 out of 194 was obtained in this study after scoring was used to evaluate the results in accordance with the treatment planning research guidelines.

### Statistical analysis

Two-tailed t-tests were conducted for each treatment plan endpoint using RapidPlan and PlanIQ as well as for the calculated MU. The same analysis was applied to the point-dose and dose-distribution validation items. All statistical analyses were performed using the *t*-test function in Excel, with *p* < 0.05 considered to indicate a significant difference.

## Results

### Results of treatment planning using RapidPlan and PlanIQ

Table 4 provides the results of the treatment planning using both RapidPlan and PlanIQ. For each endpoint, the maximum value is double-underlined, the minimum value is single-underlined, and the statistically significant *p*-values are shown in bold. The reference treatment plan was evaluated across nine items: PTV-R (D2%, D50%, and D98%), bowel (V40 Gy, V60 Gy, V70 Gy, and V75 Gy), bladder (V40 Gy and V65 Gy), and HI for PTV-R. Here, D2% indicates the maximum dose (lower values are preferable), D98% represents the minimum dose (higher values are preferable), and HI reflects the dose uniformity in PTV-R (lower values are better).

**Table 4:** Results of 12 dosimetric assessments comparing treatment plans developed using RapidPlan and PlanIQ.

Structure		Plan	Patient 1	Patient 2	Patient 3	Patient 4	Patient 5	Patient 6	Patient 7	Patient 8	Patient 9	Patient 10	Patient 11	Patient 12	Mean ± SD	*p*-value
PTV-R	D2% [Gy]	RapidPlan	81.77	81.02	81.84	82.20	81.68	82.09	82.36	81.78	82.27	82.19	82.19	82.71	82.01 ± 0.41	**0.031**
		PlanIQ	81.19	81.92	81.23	81.40	81.45	81.57	81.14	81.51	82.37	81.86	81.86	82.13	81.64 ± 0.37	
	D50% [Gy]	RapidPlan	80.63	80.08	80.78	80.97	80.51	80.50	81.32	80.55	80.82	80.85	80.85	81.45	80.78 ± 0.35	0.187
		PlanIQ	80.20	80.76	80.36	80.33	80.64	80.78	80.23	80.35	81.05	80.65	80.65	81.02	80.58 ± 0.28	
	D98% [Gy]	RapidPlan	76.53	76.68	76.39	76.29	76.35	76.56	76.18	76.54	76.03	76.20	76.20	76.06	76.33 ± 0.20	0.165
		PlanIQ	76.68	76.23	76.55	76.54	76.42	76.65	76.66	76.50	75.92	76.48	76.48	76.10	76.44 ± 0.23	
	HI	RapidPlan	0.065	0.054	0.067	0.073	0.066	0.069	0.076	0.065	0.077	0.074	0.074	0.082	0.070 ± 0.007	**0.047**
		PlanIQ	0.056	0.070	0.058	0.060	0.062	0.061	0.056	0.062	0.080	0.067	0.067	0.075	0.065 ± 0.007	
Rectum	V40 Gy[%]	RapidPlan	23.96	32.30	30.00	31.30	28.65	25.56	39.72	32.79	34.91	29.86	29.82	33.18	31.00 ± 3.97	**0.034**
		PlanIQ	29.61	32.30	34.66	30.27	33.24	29.71	34.79	33.91	41.81	35.34	35.35	31.88	33.57 ± 3.20	
	V60 Gy[%]	RapidPlan	12.26	14.60	13.17	15.06	12.15	12.11	17.17	15.25	15.54	12.60	12.59	16.28	14.06 ± 1.72	**0.003**
		PlanIQ	15.59	14.63	16.36	15.02	14.76	13.76	15.93	17.34	21.88	17.61	17.55	17.86	16.52 ± 2.06	
	V70 Gy[%]	RapidPlan	6.917	6.004	5.563	7.724	5.325	6.103	8.724	6.864	6.281	4.628	4.652	8.443	6.436 ± 1.29	**0.002**
		PlanIQ	10.44	6.812	7.298	7.772	6.631	7.040	7.566	8.945	10.82	7.694	7.747	10.39	8.263 ± 1.44	
	V75 Gy[%]	RapidPlan	3.461	2.195	1.418	3.091	1.390	2.663	3.940	1.835	1.361	0.665	0.737	3.581	2.195 ± 1.09	**0.019**
		PlanIQ	6.930	2.264	2.154	3.073	1.938	2.990	2.688	3.452	4.167	1.686	1.713	5.505	3.213 ± 1.54	
Bladder	V40 Gy[%]	RapidPlan	17.48	15.45	19.59	16.22	21.57	18.10	12.73	46.70	46.19	43.25	45.91	36.65	28.32 ± 13.4	0.836
		PlanIQ	19.26	17.66	23.40	18.23	23.27	19.00	15.45	40.13	38.59	43.18	43.23	35.77	28.10 ± 10.6	
	V65 Gy[%]	RapidPlan	7.856	4.247	5.629	4.696	5.176	7.925	12.81	25.01	23.78	25.54	25.60	18.70	13.91 ± 8.72	0.620
		PlanIQ	8.191	5.848	7.007	6.360	6.620	9.081	3.580	22.93	22.78	25.59	25.64	18.03	13.47 ± 8.34	

PlanIQ achieved greater dose reductions for the PTV-R endpoints in the high-dose regions (D2% and D50%) in 10 and eight of the 12 patients, respectively, compared with RapidPlan. Conversely, PlanIQ delivered a higher dose in nine of 12 patients (D98 %). The statistical analysis revealed a significant difference in D2% (*p* = 0.031), whereas D50% (*p* = 0.187) and D98% (*p* = 0.165) did not show significant differences. PlanIQ provided more uniform results in 10 of the 12 patients for the HI for PTV-R, with a significant difference (*p* = 0.047).

RapidPlan provided greater rectal dose reductions for V40 Gy in nine of 12 patients, and for V60, V70, and V75 Gy in 10, 11, and 11 of 12 patients, respectively. Statistical analysis showed significant differences for V40 Gy (*p* = 0.034), V60 Gy (*p* = 0.003), V70 Gy (*p* = 0.002), and V75 Gy (*p* = 0.019), indicating that the RapidPlan achieved a better dose reduction to the rectum in all patients.

RapidPlan reduced the bladder irradiation dose to V40 Gy in seven of 12 patients and to V65 Gy in eight of 12 patients. However, no significant differences were observed between RapidPlan and PlanIQ at V40 (*p* = 0.836) and V65 Gy (*p* = 0.620).

Table 5 provides the calculated MU results for the treatment plans using RapidPlan and PlanIQ. PlanIQ demonstrated lower MU values than RapidPlan in 11 of 12 patients, indicating reduced plan complexity with a significant difference at *p* = 0.002.

**Table 5:** Results of the monitor unit calculations for treatment plans using RapidPlan and PlanIQ.

	Plan	Patient 1	Patient 2	Patient 3	Patient 4	Patient 5	Patient 6	Patient 7	Patient 8	Patient 9	Patient 10	Patient 11	Patient 12	Mean ± SD	*p*-value
Total[MU]	PlanIQ	581.0	642.0	638.0	663.0	620.0	603.0	629.0	644.0	710.0	653.0	673.0	689.0	645.4 ± 35.8	**0.002**
	RapidPlan	697.0	712.0	710.0	664.0	720.0	669.0	648.0	694.0	742.0	678.0	682.0	688.0	692.0 ± 26.3	

### Results of dose verification for treatment plans with RapidPlan and PlanIQ

**Fig. 1:**
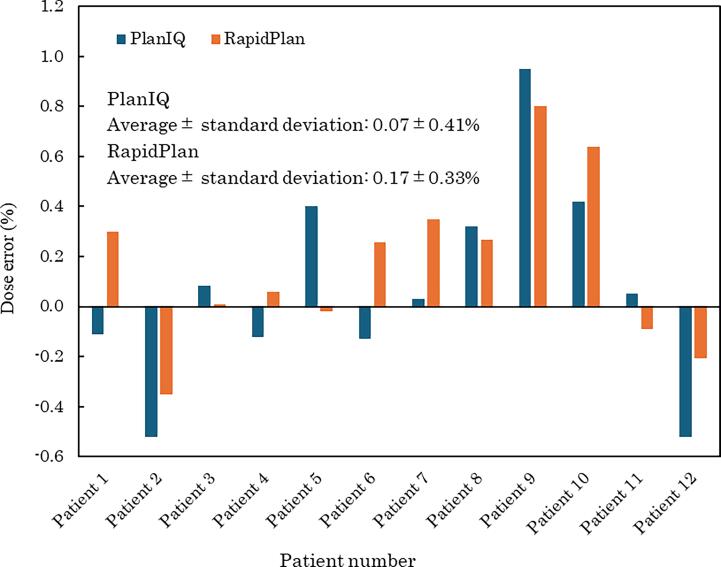
Results of point dose verification for treatment plans using RapidPlan and PlanIQ. The figure displays the mean and standard deviation of the dose differences between treatment planning system calculated doses and doses measured with an ionization chamber dosimeter.

Fig. 1 illustrates the results of point-dose verification, showing the mean and standard deviation of dose differences between the TPS-calculated doses and those measured using an ionization chamber dosimeter. The difference between the TPS and measured values was smaller for RapidPlan than for PlanIQ in seven of the 12 patients. The mean difference between the TPS and measured values for both plans was within 0.1 %. The smallest differences were observed in patients 3, 4, and 11, whereas the largest differences were observed in patients 2, 9, and 10. However, statistical analysis revealed no significant difference at *p* = 0.189.

Table 6 displays the γ-analysis results for dose distribution validation. Patients with the largest differences in point dose verification (patients 2, 9, and 10) are doubly underlined, whereas those with the smallest differences (patients 3, 4, and 11) are single-underlined. Table 7 summarizes the statistical analysis results for both the point dose and dose distribution validation.

**Table 6:** Results of the γ-analysis for dose distribution validation of treatment plans using RapidPlan and PlanIQ.

Patient number	PlanIQ			RapidPlan		
	1 mm/1%	2 mm/2%	2 mm/3%	1 mm/1%	2 mm/2%	2 mm/3%
Patient 1	95.3	100.0	100.0	93.8	100.0	100.0
Patient 2	94.4	99.8	100.0	92.6	99.8	100.0
Patient 3	91.4	100.0	100.0	94.6	100.0	100.0
Patient 4	87.5	99.8	100.0	89.0	100.0	100.0
Patient 5	91.9	100.0	100.0	87.0	100.0	100.0
Patient 6	91.5	100.0	100.0	93.8	99.8	100.0
Patient 7	90.7	99.8	100.0	92.0	99.6	100.0
Patient 8	90.4	99.8	100.0	86.7	99.5	100.0
Patient 9	92.1	100.0	100.0	88.8	100.0	100.0
Patient 10	88.5	99.8	100.0	89.6	97.3	100.0
Patient 11	88.5	99.8	100.0	90.9	99.8	100.0
Patient 12	91.7	99.6	100.0	92.9	99.8	100.0
Average	91.2	99.9	100.0	91.0	99.6	100.0
Standard deviation	2.3	0.1	0.0	2.7	0.8	0.0

**Table 7:** Statistical analysis of dose validation results for treatment plans using RapidPlan and PlanIQ across different endpoints.

Verification	Criteria	*p*-value
Point verification		0.189
γ-analysis	1 mm/1%	0.829
	2 mm/2%	0.291
	2 mm/3%	0.12

For γ-analysis, the mean γ-pass rates for 1 mm/1%, 2 mm/2%, and 2 mm/3% were 91.2–100 % for PlanIQ and 91.0–100 % for RapidPlan, with PlanIQ showing slightly higher mean values. The γ-pass rates for 2 mm/2% in patients 2, 9, and 10 (with the largest point dose differences) and patients 3, 4, and 11 (with the smallest differences) were 99.8–100.0 % for PlanIQ and 99.7–100.0 % for RapidPlan. The standard deviations of the γ-pass rates were 0.0–2.3 % for PlanIQ and 0.0–2.7 % for RapidPlan, indicating greater variability with RapidPlan. Statistical analysis of the γ-analysis results showed no significant differences, with *p*-values ranging from 0.120 to 0.829.

This study has a notable limitation: the RapidPlan model was constructed using treatment plans created with PlanIQ as references. This approach inherently aligns the RapidPlan model with PlanIQ, potentially limiting the independence of the evaluation. While PlanIQ simplifies the model-building process by providing consistently high-quality plans and eliminating the need for extensive patient selection, it may also reduce variability, thus making direct comparisons less generalizable.

Additionally, treatment planning with a RapidPlan model constructed without PlanIQ has already been widely studied, and such an investigation was beyond the scope of this study. Future research should consider comparing models developed independently of PlanIQ to further assess the robustness and versatility of the RapidPlan approach.

## Discussions

This study evaluated the treatment plans developed using RapidPlan and PlanIQ, focusing on dose uniformity to the PTV-R and dose reduction for OARs. The accuracy of the dose calculation was further assessed through the dose validation of the treatment plans.

The results provided in Table 4 indicate that treatment planning with PlanIQ resulted in better dose uniformity for the PTV-R, whereas RapidPlan was more effective at reducing doses to the OARs. This finding aligns with a previous study by Masumoto et al. who demonstrated that RapidPlan models prioritized dose reduction to OARs [8]. In contrast, the FDVH tool in PlanIQ tends to emphasize uniform and intensive irradiation of PTV-R [8].

Our study utilized equipment, X-ray energy, and dose calculation algorithms which were different from those used by Masumoto et al. Specifically, while their study used the Acuros XB, our study used the AAA. Hirashima et al. reported that the effectiveness of RapidPlan is independent of treatment equipment, energy, and MLC type [15]. Additionally, Zhenia et al. reported that significant differences between AAA and Acuros XB were observed only in targets with dense bone, with no notable differences in the OARs [16]. Given these differences, our findings suggest that both RapidPlan and PlanIQ exhibit similar trends in dose reduction for OARs and target uniformity despite variations in treatment equipment, X-ray energy, and dose calculation algorithms.

The results provided in Table 5 reveal that treatment planning with RapidPlan resulted in higher calculated MUs and increased the complexity of the intensity modulation compared with PlanIQ. Previous research by Kubo et al. indicated that higher MU values are associated with an increased complexity of intensity modulation [17]. However, Fig. 1 shows no significant difference in point-dose verification between the treatment plans using RapidPlan and PlanIQ. On average, the PlanIQ treatment plans exhibited a smaller difference between the TPS-calculated doses and measured values.

Table 6 further supports this observation, showing that PlanIQ-based treatment plans generally had higher mean values and smaller standard deviations in dose distribution validation. This might be attributed to the fact that the focus of RapidPlan on reducing doses to OARs resulted in increased complexity and variability in dose uniformity to the target. Masumoto et al. indicated that RapidPlan models prioritize OAR dose reduction [8]. While KBP-based treatment plans are known for their efficiency in reducing OAR doses, this often leads to increased plan complexity and variations in dose verification results [18]. Additionally, although different treatment planning devices and dose calculation algorithms were used compared with the study by Phillip et al. the trends observed in our study regarding increased complexity and OAR dose reduction were consistent with those reported in the literature [18].

Patients 2, 9, and 10 exhibited the largest differences in point dose verification, with discrepancies ranging from −0.58 to 0.93 % for PlanIQ and −0.35 to 0.88 % for RapidPlan. Despite these differences, the standard deviations for the γ-analysis (2 %/2 mm) were 0.1 % for PlanIQ and 0.8 % for RapidPlan, indicating less variation with PlanIQ. This finding contrasts with the results of Stambaugh et al. who reported a γ pass rate of 92.8 ± 3.9 % (range 89.5–99.2 %) using a 2 %/2 mm criterion [19]. Thus, our results suggest that the variability in dose distribution validation was smaller for both treatment plans. In addition, PlanIQ generally demonstrated better agreement rates for dose verification than did RapidPlan.

## Conclusions

This study observed that treatment planning using RapidPlan was more effective in reducing the doses of the OARs. Conversely, PlanIQ demonstrated superior performance in terms of treatment planning complexity and dose uniformity to the target. Although dose verification showed no significant differences between the two treatment plans, the PlanIQ-based plans generally exhibited better dose-distribution matching. These findings suggest that the choice of a treatment planning tool should align with the primary objectives of the facility’s treatment goals.

Informed Patient Consent.

Written informed consent for the inclusion of this information in this case report has been obtained from the patient or, where applicable, from the patient's parent, guardian, or power of attorney, and they have approved the publication of this information.

Data statement.

The datasets used and/or analyzed during the current study are available from the corresponding author on reasonable request.

## Funding

No funding was received specifically for this study.

## Declaration of competing interest

The authors declare that they have no known competing financial interests or personal relationships that could have appeared to influence the work reported in this paper.
